# Synchronous Primary Gallbladder and Colon Adenocarcinoma: A Case Report and Systematic Literature Review

**DOI:** 10.7759/cureus.69092

**Published:** 2024-09-10

**Authors:** Paraskevi Dedopoulou, Nikiforos Rodis, Charalampos Lampropoulos, Konstantina Soultana Kitsou, Nikolaos Mpogiatzopoulos, Ioannis Kehagias

**Affiliations:** 1 Department of Surgery, General University Hospital of Patras, Patras, GRC; 2 Intensive Care Unit, General University Hospital of Patras, Patras, GRC

**Keywords:** adenocarcinoma, colorectal cancer, gallbladder cancer, multiple primary malignancies, synchronous malignancies

## Abstract

Synchronous primary malignancies, defined as two or more primary malignancies diagnosed simultaneously or within six months, are uncommon and present unique diagnostic and therapeutic challenges. Synchronous primary adenocarcinoma of the gallbladder and colon is particularly rare. We report a case of a 48-year-old female presenting with persistent right upper abdominal pain. Laboratory tests and imaging studies initially suggested xanthogranulomatous cholecystitis. However, subsequent laparoscopic cholecystectomy and pathological examination revealed a moderately differentiated adenocarcinoma of the gallbladder (pT2bN1M0). Further staging with CT and PET-CT scans identified a suspicious mass in the transverse colon, confirmed by colonoscopy and surgical resection as well-differentiated adenocarcinoma of the transverse colon (pT3N0M0). Immunohistochemistry and genetic profiling of both tumors indicated distinct primary origins without loss of mismatch repair (MMR) protein expression. The patient underwent additional liver resection, lymph node dissection, and right extended hemicolectomy. She is currently undergoing further staging and awaiting chemotherapy. A review of English-language literature revealed eight reported cases of synchronous primary gallbladder and colorectal cancer and a total of 13 with synchronous primary malignancy of other organs. Such cases are rare and diagnostically complex cases. Common factors contributing to multiple primary malignancies (MPM) include genetic predispositions, previous cancer treatments, and lifestyle factors such as smoking and alcohol consumption. This case underscores the importance of thorough investigation and prompt treatment in patients suspected of having MPM. Advances in diagnostic imaging and molecular profiling are crucial for early detection and tailored therapeutic strategies. Standardized guidelines for managing synchronous cancers are needed to improve patient outcomes.

## Introduction

Multiple primary malignancies (MPM) are defined as the occurrence of two or more primary malignancies with distinct histological origins in a single individual, excluding metastasis or recurrence of a primary tumor. The concept of MPM was first described by German surgeon Theodor Billroth in 1889, who documented cases of patients developing more than one distinct primary cancer [1]. A subsequent report in 1921, analyzing 3,000 cases, found that 4.7% of patients had multiple growths [2].

MPM can be classified based on the timing of diagnosis: synchronous malignancies are those diagnosed simultaneously or within six months, while metachronous malignancies are diagnosed more than six months apart [3].

The incidence of MPM in cancer populations varies between 2.4% and 8%, with up to 17% developing multiple primaries within 20 years of follow-up ​[2].

Synchronous primary malignancies of the gallbladder and colon or rectum are exceedingly rare, with only eight cases reported in the English literature [4-11]. The rarity and atypical presentation of these synchronous malignancies pose significant diagnostic and therapeutic challenges for clinicians. Accurate diagnosis is crucial, as misdiagnosis can adversely affect the prognosis. Gallbladder cancer, though rare, accounts for 50% of hepatobiliary cancers and is the fifth most common gastrointestinal cancer. Conversely, colon cancer is the second leading cause of cancer-related deaths worldwide, responsible for over 930,000 deaths annually ​[12,13]. The coexistence of synchronous primary cancers of the gallbladder and colon is exceptionally uncommon.

Herein, we present the case of a 48-year-old female who presented with abdominal pain and was subsequently diagnosed with moderately differentiated adenocarcinoma of the gallbladder (pT2bN1M0) and well-differentiated adenocarcinoma of the transverse colon (pT3N0M0), with no loss of MMR protein expression in either tumor. Our aim is to underscore the importance of accurate diagnosis and the potential for synchronous MPM in patients diagnosed with a malignancy. Early-stage diagnosis is critical for the patient and essential for selecting the appropriate therapeutic strategy.

## Case presentation

A 48-year-old female presented to the emergency department with a seven-day history of persistent, dull abdominal pain localized to the right upper quadrant. The patient did not report experiencing any other symptoms, including gastrointestinal symptoms. Laboratory investigations revealed a white blood cell count of 15.69 K/μL, C-reactive protein (CRP) of 0.25 mg/dL, and an elevated amylase level of 506 IU/dL. An upper abdominal ultrasound demonstrated increased gallbladder wall thickness and the presence of a tissue mass. A subsequent abdominal computed tomography (CT) scan revealed a heterogeneous mass with peripheral contrast enhancement adjacent to the gallbladder, suggesting a possible confined rupture or abscess. Empiric antibiotic therapy was initiated, and magnetic resonance cholangiopancreatography (MRCP) indicated mild dilation of the common and cystic ducts, consistent with xanthogranulomatous cholecystitis.

**Figure 1:**
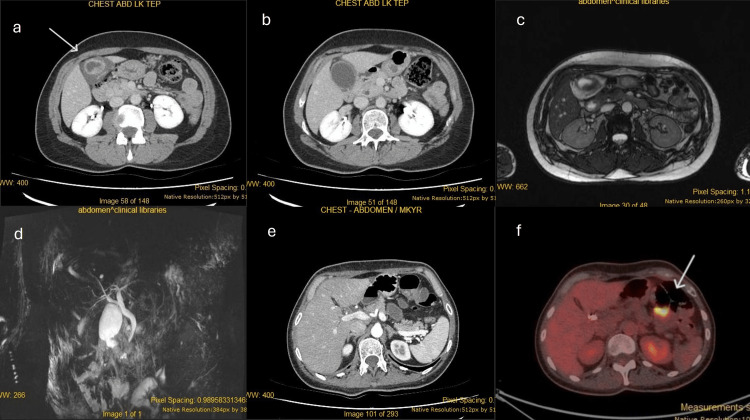
Imaging Pictures a, b: initial presentation CT scan; pictures c, d: MRI and MRCP scans; picture e: post-cholecystectomy dual-phase CT; picture f: PET CT scan post-cholecystectomy

The patient self-discharged but returned two weeks later due to persistent symptoms. Cancer biomarker analysis revealed carcinoembryonic antigen (CEA) levels at 4.23 ng/mL (normal range: 0.0-10.0 ng/mL) and CA 19-9 levels at 12.34 U/mL. The patient underwent laparoscopic cholecystectomy, with pathological examination revealing a stage pT2b N1 adenocarcinoma of the gallbladder, adjacent to the hepatic side, with no evidence of gallstones. A dual-phase contrast CT scan for staging purposes showed a suspicious lesion in the transverse colon and mild large bowel distention, prompting a positron emission tomography (PET) CT scan (Figure 1). The PET scan revealed fluorodeoxyglucose (FDG) uptake by a mass in the transverse colon (standardized uptake value - SUVmax: 10.3), which was confirmed by colonoscopy.

One-month post-cholecystectomy, the patient underwent microwave ablation and anatomical resection of liver sections IVb and V, hepatoduodenal ligament lymph node dissection, and a right extended hemicolectomy due to a mass near the splenic flexure of the transverse colon. The patient had an uneventful recovery and was discharged from hospital. Pathological examination of the resected specimen revealed well-differentiated adenocarcinoma of the large bowel, with 43 lymph nodes examined and no metastasis, staging the disease at pT3N0. Currently, the patient is undergoing further staging with magnetic resonance imaging (MRI) and is awaiting additional chemotherapy treatment under oncologic supervision.

Pathology 

Immunohistochemical analysis of the colonic adenocarcinoma revealed the following profiles: CK7-, CK20+, CDX2+, and SATB2+. Re-examination of the gallbladder adenocarcinoma specimen established a different immunohistochemical profile: CK7+, CK20-, CDX2+, and SATB2-. These distinct profiles confirmed that the colonic tumor and gallbladder tumor originated independently, diagnosing two separate primary adenocarcinomas.

Further molecular analysis of the colonic adenocarcinoma identified a G12X mutation in the KRAS gene, with no mutations detected in BRAF or NRAS genes. Both adenocarcinomas exhibited intact nuclear expression of mismatch repair (MMR) proteins, indicating no loss of MMR protein expression.

## Discussion

Literature review 

**Figure 2:**
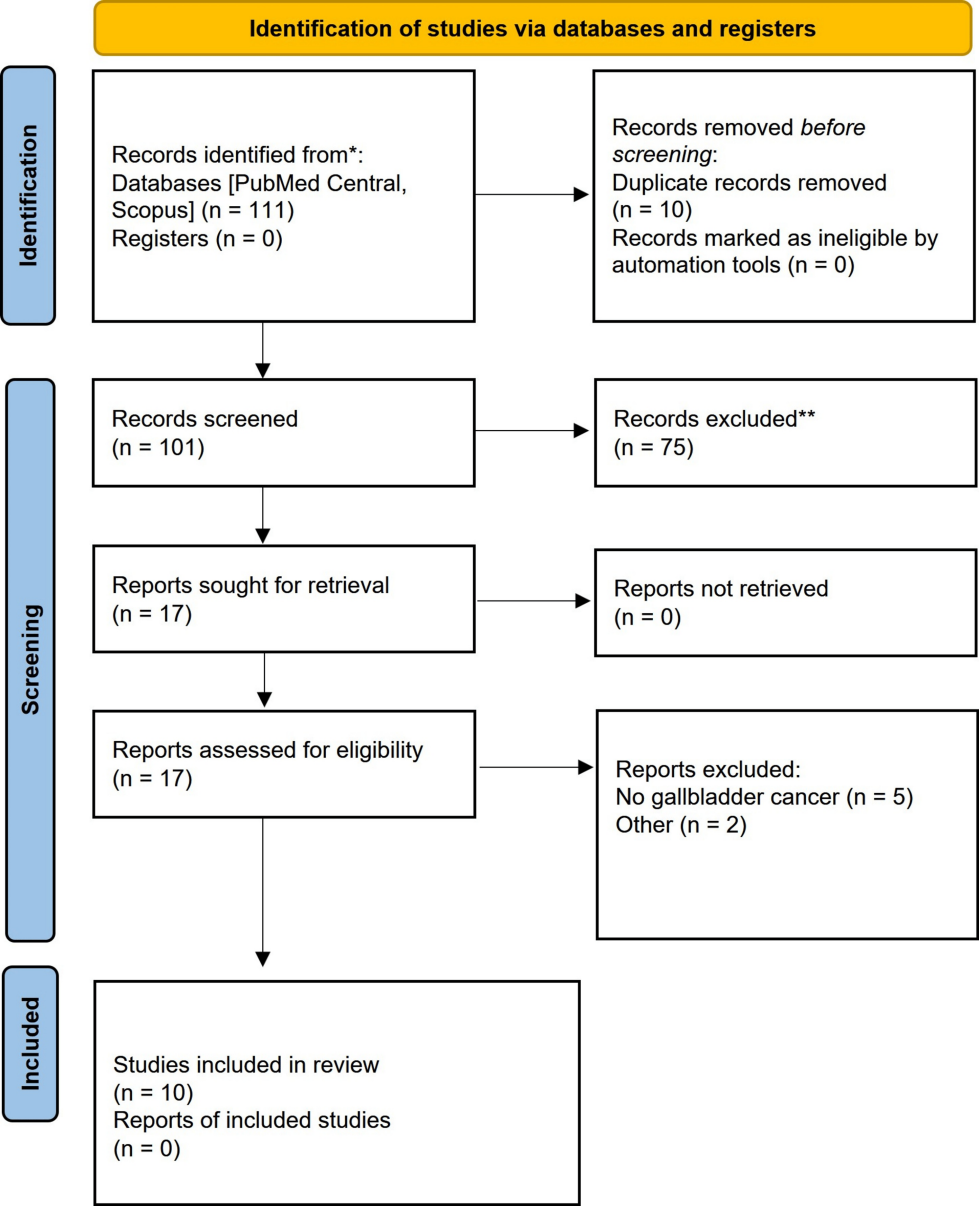
PRISMA study flow chart PRISMA: Preferred Reporting Items for Systematic Reviews and Meta-Analyses

We searched the PubMed database (National Library of Medicine, Bethesda, MD, USA) and Scopus in May 2024 and June 2024 for published human studies using the terms “synchronous”, “cancer”, “gallbladder”, and “colon” in all possible combinations. All steps followed the Preferred Reporting Items for Systematic Reviews and Meta-Analyses (PRISMA) (1) best-practice guidelines for systematic reviews (Figure 2) [14]. 

The search keywords were determined through the acronym PICOS: P (population): patients with synchronous gallbladder and colon or rectal cancer; I (intervention): operation; C (comparison): existing treatments; Ο (outcome): survival rates, quality of life, recurrence rates; S (studies): cohort studies and case reports.

The Medical Subject Headings (MeSH) terms in English used were as follows: ((synchronous) OR (simultaneous)) AND ((cancer) OR (adenocarcinoma) OR (neoplasm)) AND (gallbladder) AND ((colon) OR (colonic) OR (rectum) OR (rectal)).

Inclusion/exclusion criteria

We applied a series of inclusion/exclusion criteria as follows. Articles were included if they: (i) used case reports and case series and (ii) included studies involving patients diagnosed with synchronous primary adenocarcinoma of the gallbladder and colon. Studies focusing solely on either gallbladder adenocarcinoma or colon adenocarcinoma without addressing the synchronous occurrence of both were excluded. We removed also articles that were published in a language other than English. Studies without available full text were excluded from further analysis.

This initial search resulted in 111 articles, of which 44 were from PubMed and 67 from Scopus. After removing duplicates (n = 10), 101 articles remained for screening and eligibility. 

Extraction and analysis

Study selection was conducted by two reviewers (DP, RN) independently. The eligibility criteria were first used to exclude titles, then abstracts, and finally full articles. The bibliography of the included studies was also screened by hand. The selection was performed using Microsoft Office Excel version 2406 (Microsoft Corporation, Redmond, USA). A third reviewer (LC) was consulted if there was no consensus between the two reviewers. Data extraction also was conducted by two reviewers (KK, MN) independently and discrepancies were resolved through consensus.

Screening of the title and abstracts resulted in removing 75 papers, leaving 17 articles. All were successfully retrieved. Further applying eligibility to the full text left a final sample of 10 studies for inclusion in the systematic review study.

From the 10 articles, we extracted the main data on the study, such as the age of the patient, the sex, the location of the tumors, the histopathology, the stage (TNM), and the tumor markers. These data were analyzed and classified and are presented in Tables 1-2).

**Table 1:** Synchronous multiple primary malignancies involving the gallbladder and colon or the rectum F: Female; M: Male; AC: Ascending Colon; TC: Transverse Colon; GB: Gallbladder; VA: Vermiform Appendix; SC: Sigmoid Colon; R: Rectum; CEA: Carcinoembryonic Antigen

Author	Year	Age	Sex	Location	Histopathology	Stage (TNM)	Tumor markers
Schmid et al. [4]​	1988	79y	F	Transverse colon (TC) Appendix (VA), Gallbladder (GB).	TC: Moderated differentiated mucinous adenocarcinoma. VA: non-invasive adenocarcinoma. GB: Well-differentiated adenocarcinoma.	TC: pT3, N0, M0; GB:pT2, N0, M0	
Tamura et al. [5]	2003	70y	M	Sigmoid Colon (SC), Gallbladder (GB), Stomach.	SC: Well-differentiated adenocarcinoma. Stomach: well-differentiated adenocarcinoma. GB: Papillary adenocarcinoma.	SC: Stage I. GBC: Stage I. Stomach: T1, N0, M0 (Stage II A)	CEA: 2.4 ng/mL; CA-19:23 ng/mL
Sakellaridis et al.​ [6]	2005	72y	F	Rectum (R), Galbladder (GB).	R: Middle differentiated adenocarcinoma. GB: middle differentiated adenocarcinoma.	RC: T2, N0, M0; Stage II; GBC: T2, N0, M0; Stage II	CEA: 15.9 ng/mL
Gupta et al.​ [7]	201	40y	F	Rectum (R), Gallbladder (GB).	R: Moderately differentiated adenocarcinoma. GB: Moderately differentiated adenoma.	RC: pT3, N0, M0 (Stage IIA). GBC: pT2, N0, M0 (Stage II)	CEA:19 ng/mL; CA19-9: Normal
Alruwaii et al. ​[8]	2014	30y	F	Sigmoid Colon (SC), Gallbladder (GB).	SC: Moderately differentiated adenocarcinoma. GB: In situ adenocarcinoma.	SC: pT4b, N1, M0. GB: Tis, N0, M0	
Chablou et al. [9]	2021	59y	F	Sigmoid colon (SC), Gallbladder (GB).	SC: Well-differentiated infiltrating adenocarcinoma, lymph node metastases. GB: large cell high-grade neuroendocrine carcinoma (NET).	SC: pT3N1b; GB: G3 NET	CA19-9, CA125, CEA: Negative
Galanis et al. [10]	2023	75y	F	Sigmoid colon (SC), Gallbladder (GB).	SC: Moderately differentiated adenocarcinoma. GB: Poorly differentiated adenocarcinoma.	SC: pT3, N2, M0; GB: pT2b, N0, M0	
Dash et al.​ [11]	2023	59y	M	Ascending colon (AC), Gallbladder (GB).	AC: invasive adenocarcinoma. GB: intestinal type adenocarcinoma.	AC: pT1N0MO. GB: pT3N0M0	
Present case	2024	48y	F	Transverse colon (TC), Gallbladder (GB).	TC: well-differentiated adenocarcinoma. GB: Middle differentiated adenocarcinoma.	TC: pT3, N0, MO. GB: pT2b, N1, M0	CEA: 4.23 ng/mL; CA19-9: 12,34 U/mL

**Table 2:** Synchronous multiple primary malignancies involving the gallbladder and other organs F: Female; M: Male; TC: Transverse Colon; GB: Gallbladder; SC: Sigmoid Colon; R: Rectum; CEA: Carcinoembryonic Antigen

Author	Year	Age	Sex	Location	Histopathology	Stage (TNM)	Tumor markers
Dash et al. [11]​	2023	48y	F	Breast Gallbladder	Breast: Invasive duct carcinoma. GB: Invasive Adenocarcinoma.	Breast: pT1, N2, M0. GB: pT2, N1, M0	ER, PR: positive
	2023	56y	F	Renal Gallbladder	Renal: clear cell type. GB: Invasive adenocarcinoma.	Renal: Grade 2. GB: pT3, N1, M0	
Gaurav et al.​ [15]	2011	65y	F	Renal Gallbladder	Transitional cell Carcinoma (TCC). GB: Adenocarcinoma.		
Zhou et al.​ [16]	2017	46y	M	Stomach Gallbladder	Stomach: poorly differentiated carcinoma. GB: Adenocarcinoma.	Grade II	CEA: 8.79 ng/mL
	2017	80y	F	Stomach Gallbladder	Stomach: gastric adenocarcinoma with local innovation. GB: adenocarcinoma.	Stomach: Stage III. GB: Stage III	CEA: 4.06 ng/mL. CA 19-9: 68.84 U/mL

Results

Ten authors have reported cases involving 13 patients with MPM, including gallbladder cancer [4-11,15,16]. Of these, 12 patients had two primary cancers, while one had three distinct primary cancers [5]. The majority of these cases involved malignancies in other parts of the digestive tract, such as the colon or rectum, stomach, and vermiform appendix. Additionally, two cases involved renal primary cancer, and one involved breast cancer [11].

The mean age of the patients was 61 years, and the majority (10 of 13) were female. Most had no significant medical history, except for one patient who had hypertension and epilepsy. Seven patients presented with vague abdominal symptoms, such as dull upper abdominal pain, prior to the diagnosis of gallbladder cancer. Some patients with concurrent primary colon or rectal cancer exhibited symptoms such as hematochezia and constipation. Tumor markers CEA and CA-19 were mentioned in four cases of synchronous gallbladder and colorectal cancer.

In one case with concurrent gastric adenocarcinoma, CEA was 2.4 ng/mL, and CA-19 was 23 ng/mL [5]. In another case of synchronous gallbladder and rectal cancer, CEA was 15.9 ng/mL [6]. In the third case, CEA was 19 ng/mL, and CA-19 was within the normal range [7]. In a fourth case, CA19-9, CA125, and CEA were all within normal ranges [9].

Regarding the treatment of gallbladder disease, six patients underwent radical or simple cholecystectomy with hepatoduodenal lymph node dissection, with or without hepatic resection, in addition to the treatment of concurrent primary cancer. This included procedures such as endoscopic mucosal resection of a sigmoid tumor, partial gastrectomy, and radical nephrectomy [5,10,11,16]. Seven patients underwent simple cholecystectomy [6-8,10,11,16].

Table 2 summarizes cases of synchronous primary gallbladder and colorectal cancer, along with our presented case. 

Discussion

The incidence of MPM has been increasing, with reported occurrence rates ranging from 0.7% to 11.7%. This rise is likely attributable to advancements in diagnostic imaging and other diagnostic techniques. Most MPM involve the respiratory, gastrointestinal, or genitourinary system ​[17].

MPM involves a complex interplay of risk factors, including genetic predispositions, environmental exposures, host factors, prior malignancies or cancer treatments, and immunosuppressive conditions. Understanding these risk factors is crucial for the early detection, prevention, and management of MPM [18]​.

Genetic predispositions play a pivotal role in MPM pathogenesis. Inherited cancer syndromes, such as Lynch syndrome, predispose individuals to a range of cancers at a younger age compared to the general population. Mutations in DNA repair genes, including MLH1, MSH2, MSH6, PMS2, and EPCAM, impair DNA repair mechanisms and contribute to carcinogenesis ​[19,20]. Additionally, mutations in BRCA1 and BRCA2 are linked to heightened risks of breast and ovarian cancers [21,22]. Environmental and host factors also contribute significantly to MPM development. Carcinogenic influences such as smoking, alcohol consumption, exposure to ionizing radiation, organic and inorganic chemicals, obesity, and hormonal therapy are implicated. For example, alcohol consumption is associated with approximately 3.6% of all cancers ​[23]. Prior cancer treatments, including radiation and chemotherapy, can increase the risk of subsequent malignancies [24]. Travis et al. documented an elevated risk of leukemia following platinum-based chemotherapy for ovarian cancer [25-27]. In our case, the patient had no family history of cancer and did not smoke or consume alcohol.

Immunosuppression is another critical factor, with immunodeficient individuals at increased risk for specific cancers. Immunodeficiency syndromes are linked to a higher incidence of non-Hodgkin lymphoma, while immunosuppressive therapy further raises the risk for non-Hodgkin lymphoma, Kaposi sarcoma, and squamous cell skin cancer. Human papillomavirus (HPV) is a known etiological factor for cervical, anal, and oropharyngeal cancers [24,28,29].

Gallbladder cancer, the most prevalent biliary tract malignancy, constitutes approximately two-thirds of such cases. Its late-stage diagnosis is often due to its anatomical location and vague symptoms, with only 25% of patients qualifying for surgical intervention and generally poor prognosis [12,28,30,31].​ Gallbladder cancer is more frequent in females and usually affects people 65 years old and older with an average age of diagnosis of 75 years old [32,33]. Contrary to this, our study found that the mean age of patients with MPM including the gallbladder was 61 years old, and the patients were mostly female. MPM usually appears in patients of a greater age than with a single cancer, while most of them exceed the 50-year-old threshold [17].

Chololithiasis and chronic inflammation are the most common risk factors for gallbladder cancer. High body mass index contributes to the formation of gallstones and is a known risk factor for gallbladder cancer [34]. Half of the cases of gallbladder cancer are diagnosed incidentally after cholecystectomy [24,26]. Surgery remains the primary treatment for early-stage gallbladder cancer [12,28]. Patients diagnosed with gallbladder cancer face a notably heightened risk of developing additional primary gastrointestinal cancers. These secondary malignancies can occur in the stomach, small intestine, colon, liver, and pancreas, as well as the intrahepatic and extrahepatic bile ducts [35,36].

Colorectal cancer is among the most common cancers globally and the second leading cause of cancer-related mortality, with approximately 930,000 deaths annually ​[37]. The American Cancer Society recommends screening beginning at age 45 for individuals at average risk, as incidence rates are rising among those under 50 [37]. According to Bailey et al., the incidence of colorectal cancer among individuals aged 20-34 years may increase by 90%-124% by the year 2030 [38,39]. Asia and Europe exhibit the highest incidence rates of colorectal cancer [40]. The American Joint Committee on Cancer (AJCC) TNM system is commonly used for staging, with surgical intervention being the primary treatment for early-stage cases. Prognosis is influenced by tumor invasion, nodal involvement, and metastases, with bowel obstruction and perforation generally indicating a poor outcome [41,42].

Individuals with primary colorectal cancer have a higher risk of developing a second primary cancer and exhibit a higher incidence of secondary primary cancers in the colon and rectum compared to the general population [39,43].

Additionally, patients with colorectal cancer have been found to have an increased risk of developing cancers of the small intestine, cervix, breast, kidney, thyroid, melanoma, stomach, urinary bladder, and respiratory system [44-52].

While individuals with colorectal cancer face an increased risk of developing secondary primary cancers in these organs, the risk for gallbladder cancer is reduced [35]. This makes cases of synchronous cancer of the gallbladder and colon, such as the one described, particularly rare and interesting. Worldwide, there have been 13 reported cases in the English literature, of synchronous gallbladder cancer with other primary malignancies, including eight cases involving the colon or rectum, as it was discussed above [5,6,8,10,11,16]​.

Management of MPM varies and lacks a definitive consensus, typically involving radical resection and appropriate adjuvant therapy [5,6,10]. When the neoplasms are localized, the best options might include oncologic resection plus radiation and/or chemotherapy. When there is advanced disease, treatment plans are mostly individualized and empiric, considering the nature of the neoplasm and regimen sensitivities and possible response. Response and survival rates of patients with MPM depend on the cancer type and the stage of the disease when diagnosis is made [17].

## Conclusions

In conclusion, the occurrence of synchronous primary adenocarcinomas of the gallbladder and colon presents significant diagnostic and therapeutic challenges due to its rarity. This case emphasizes the necessity for comprehensive evaluation in patients with atypical symptoms and highlights the critical role of advanced imaging and molecular profiling. Future guidelines should focus on standardizing management approaches to optimize outcomes for patients with such complex presentations.
